# NF-κB in monocytes and macrophages – an inflammatory master regulator in multitalented immune cells

**DOI:** 10.3389/fimmu.2023.1134661

**Published:** 2023-02-23

**Authors:** Marion Mussbacher, Martina Derler, José Basílio, Johannes A. Schmid

**Affiliations:** ^1^ Department of Pharmacology and Toxicology, Institute of Pharmaceutical Sciences, University of Graz, Graz, Austria; ^2^ Department of Vascular Biology and Thrombosis Research, Centre of Physiology and Pharmacology, Medical University of Vienna, Vienna, Austria; ^3^ INESC ID–Instituto de Engenharia de Sistemas e Computadores, Investigação e Desenvolvimento em Lisboa, Universidade de Lisboa, Lisboa, Portugal

**Keywords:** NF-κB – nuclear factor kappa B, monocyte – macrophage, signaling/signaling pathways, immunity, inflammation

## Abstract

Nuclear factor κB (NF-κB) is a dimeric transcription factor constituted by two of five protein family members. It plays an essential role in inflammation and immunity by regulating the expression of numerous chemokines, cytokines, transcription factors, and regulatory proteins. Since NF-κB is expressed in almost all human cells, it is important to understand its cell type-, tissue-, and stimulus-specific roles as well as its temporal dynamics and disease-specific context. Although NF-κB was discovered more than 35 years ago, many questions are still unanswered, and with the availability of novel technologies such as single-cell sequencing and cell fate-mapping, new fascinating questions arose. In this review, we will summarize current findings on the role of NF-κB in monocytes and macrophages. These innate immune cells show high plasticity and dynamically adjust their effector functions against invading pathogens and environmental cues. Their versatile functions can range from antimicrobial defense and antitumor immune responses to foam cell formation and wound healing. NF-κB is crucial for their activation and balances their phenotypes by finely coordinating transcriptional and epigenomic programs. Thereby, NF-κB is critically involved in inflammasome activation, cytokine release, and cell survival. Macrophage-specific NF-κB activation has far-reaching implications in the development and progression of numerous inflammatory diseases. Moreover, recent findings highlighted the temporal dynamics of myeloid NF-κB activation and underlined the complexity of this inflammatory master regulator. This review will provide an overview of the complex roles of NF-κB in macrophage signal transduction, polarization, inflammasome activation, and cell survival.

## 1 Introduction

Monocytes and macrophages act as immune sentinels that rapidly respond to invading pathogens and local tissue injury. Equipped with a broad repertoire of pattern recognition receptors, they can detect viral, bacterial, and fungal components and are activated by proinflammatory cytokines. Whereas monocytes preferentially traffic *via* the blood stream, macrophages are ubiquitously distributed in almost all tissues. Monocyte recruitment is initiated by a chemokine gradient that results in adhesion and transmigration. In the local tissue environment, present growth factors induce their differentiation to either macrophages or dendritic cells (1). Macrophages contribute to the clearance of pathogens, cellular debris, and infected cells. In secondary lymphoid organs, monocytes and macrophages present antigens to B and T lymphocytes enabling priming and modulation of their effector cell functions (2). Thus, macrophages play a central role in immune defense and link innate and adaptive immunity. The transcription factor NF-κβ as well as its upstream and downstream signaling molecules, which are essential for the versatile functions of monocytes and macrophages, form a highly diverse and dynamic network of signaling processes rather than a one-dimensional pathway. Important to note, that there is neither a “good” nor a “bad” monocyte/macrophage” and the same is true for NF-κB. The outcome of NF-κB activity depends on various factors such as the timing, the signaling strength and intensity, the specific microenvironment, the organ system, and the disease state (3).

## 2 The transcription factor NF-κB

**Figure 1:**
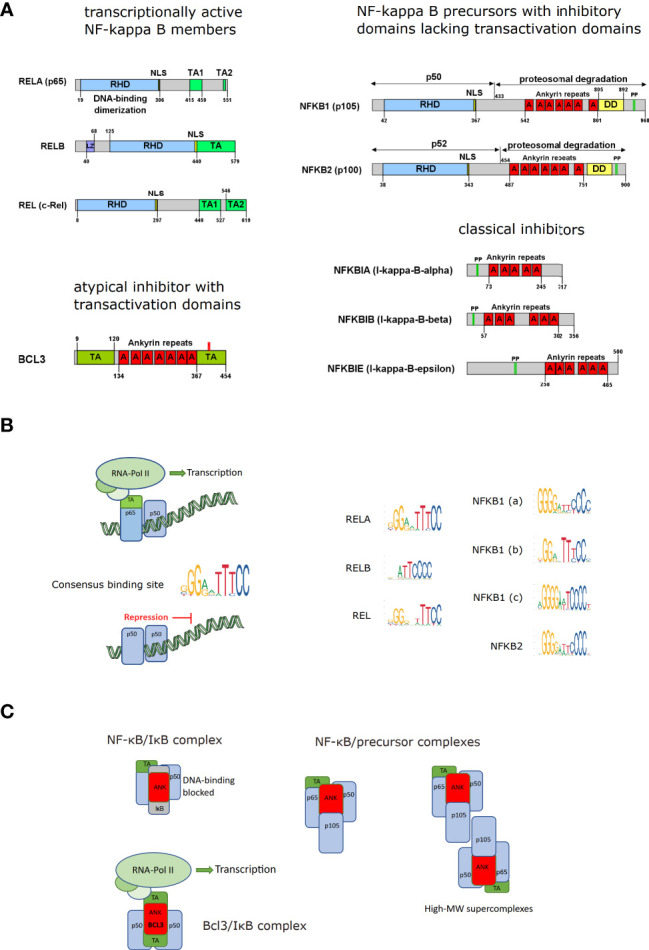
NF-κB and IκB molecules. **(A)** The domain structure of NF-κB family members is depicted with the common Rel-homology domain (RHD), nuclear localization sequences (NLS), transactivation domains (TA), leucine zippers (LZ), ankyrin repeats **(A)**, death domains (DD) and phosphorylation sites (PP) targeting poly-ubiquitination and proteasomal degradation or processing. The Gene symbol names are specified with the most common aliases (modified from (4)). The precursor proteins p105 (NFKB1) and p100 (NFKB2) give rise to the processed forms p50 and p52, respectively. **(B)** Schematic illustration of NF-κB dimers bound to DNA including active transcription factors (with transactivation domains recruiting RNA-Polymerase II [RNA Pol II]) and inactive NF-κB complexes lacking a transactivation site and acting as repressor; Consensus DNA binding motifs for the different NF-κB members: Retrieved from the JASPAR database (5) by searching for Rel homology region factors as Class and family “NF-kappaB-related factors. The height of the nucleotide reflects the degree of conservation. **(C)** NF-κB/IκB complexes and NF-κB complexes containing inhibitory precursor proteins (including high-MW super-complexes formed by binding *via* RHD and ankyrin repeats); NF-κB bound to inhibitory IκB molecules and transcriptionally active complexes between p50- or p52-homodimers bound to BCL3, an ankyrin-repeat containing IκB family member with transactivation domains, as indicated.

NF-κB, which stands for “nuclear factor regulating the antibody-kappa light chain in activated B-cells” is not a single factor but a family of transcription factors, which is built by homo- or heterodimers of two of the five family members: RelA (p65), RelB, c-Rel, p100/p52, and p105/p50 (**Figure 1A**). It has to be stated that the abbreviation is quite misleading, as in non-activated cells NF-κB is not localizing to the nucleus, but to the cytosol – and it is not only regulating the kappa light chain, but a whole plethora of target genes (4) (for an overview see (6, 7). Finally, NF-κB is not only active in B cells but basically in all human cells. The common scheme of the five NF-κB members is the so-called Rel homology domain (RHD), which is essential for dimerization and binding to double-stranded DNA with the consensus sequence (G/C)GGnnTTTCC. Variations of this canonical target sequence exist in many genes, which makes it difficult to predict by bioinformatics whether NF-κB will bind to a specific enhancer-promoter element. This is further aggravated by the fact that NF-κB molecules can sometimes bind even to half of a consensus site with significant affinity (8). Moreover, different NF-κB dimers were reported to have different binding preferences to target sequence variations, so distinct NF-κB dimers may regulate various target genes in a differential manner (8, 9) (**Figure 1B**).

An important aspect is that two of the family members (p50 and p52, as well as their respective precursors p105 and p100) do not contain a transactivation domain (TA), which is necessary for transcription *via* recruiting the RNA-polymerase complex and therefore need a binding partner with a TA domain to actively participate in gene induction. Consequently, dimers of these two family members (p50/p50; and p52/p52; theoretically possible but not observed: p50/p52 (10)) can bind to DNA but are incapable of driving transcription and can therefore act as transcriptional repressors. This has also been shown in a mouse model of a p105/p50 knockout, characterized by chronic systemic inflammation, presumably because the repressor function of p50-homodimers is lost (11). p50 and p52 are synthesized as precursor proteins (p105 and p100, respectively), holding a C-terminal ankyrin repeat domain, which has an inhibitory role as it masks at least in part the nuclear localization sequence next to the Rel-homology domain and interferes with stable binding to DNA. These inhibitory precursor proteins can form super-complexes with the other NF-κB members *via* the Rel-homology and the ankyrin-repeat domains (12) For activation of NF-κB, they have to be either degraded completely or proteolytically processed to cleave the inhibitory domains. The processed forms p50 and p52 can then be part of an active NF-κB dimer if complexed with a family member containing a transactivation domain. One of the most frequent NF-κB dimers is a complex of the mature p50 bound to RelA (p65). In non-stimulated, quiescent cells, NF-κB molecules such as the p50/p65 dimer are usually kept inactive in the cytosol by binding to an inhibitory molecule of the IκB family (inhibitors of NF-κB, IκBα, IκBβ, IκBϵ, BCL3, **Figures 1A, C**). These inhibitors contain ankyrin-repeats, very similar to the inhibitory domains of p105 and p100, which shift the steady localization of the complex to the cytosol. Nevertheless, as nearly all protein complexes, the NF-κB/IκB complex is subject to dynamic dissociation and re-association (based on k_off_ and k_on_ rates), so that a certain fraction of the proteins exists in the unbound form. In this state, both NF-κB and IκB can be recognized by the nuclear import machinery and translocated to the nucleus, so that in non-activated cells about 5% of the total NF-κB are in the nucleus (13). Yet, NF-κB and IκB proteins also contain nuclear export sequences, which is the basis for a dynamic nucleocytoplasmic shuttling (14, 15). Besides hindering an efficient import of NF-κB into the nucleus, IκB molecules prevent efficient DNA binding of NF-κB. This is also the basis for inactivation of NF-κB, as newly synthesized IκB, which is a target gene of NF-κB, translocates into the nucleus, where it binds to NF-κB, removing it from the DNA and dragging it out to the cytosol.

An atypical IκB molecule is Bcl3, which contains not only ankyrin-repeats that allow binding to p50 or p52 homodimers, but also transactivation domains. Thus, DNA binding and activation of transcription can be achieved by Bcl3/p50/p50 or Bcl3/p52/p52 complexes – a process which requires specific phosphorylation of Bcl3 (10, 16).

**Figure 2:**
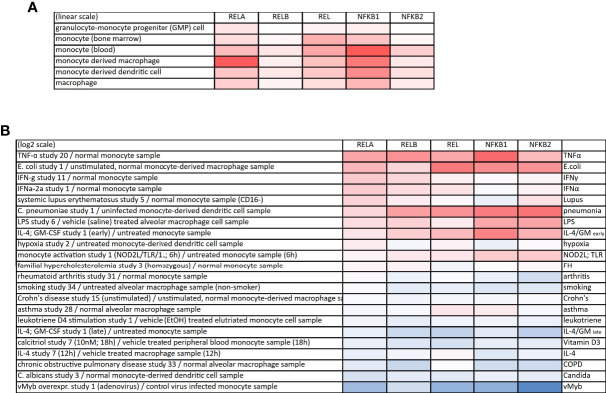
Expression of NF-κB proteins in monocytes and macrophages. **(A)** Expression of the NF-κB family members in monocytes, macrophages and monocyte-derived cells (linear scale). The gene symbols are provided: RELA (p65), RELB, REL (c-Rel), NFKB1 (p105/p50) and NFKB2 (p100/p52). **(B)** Heatmap of up- (red) or downregulation (blue) of NF-κB family members upon various perturbations. All data derived from the Genevestigator database.

All NF-κB members occur in macrophages and monocytes, however, with some predominance of RelA/p50 complexes (**Figure 2A**). Upon inflammatory activation, the expression of most NF-κB members increases, while it decreases under certain pathological conditions or after treatment with substances such as IL-4 or vitamin D_3_ (calcitriol, **Figure 2B**). On the other hand, the cellular state also influences the abundance or the posttranslational modification of NF-κB members, which has been shown, for example, for non-classical and intermediate monocytes, which express high levels of total and phosphorylated p65 when compared to classical monocytes (17). The dynamics of activation-induced NF-κB translocation are different in macrophages when compared to other cell types: Whereas in fibroblasts, NF-κB activation leads to periodic oscillations of the NF-κB dimer between the nucleus and the cytosol, macrophages show a persistent translocation of NF-κB into the nucleus (18). See (18) for more information regarding NF-κB activation and thresholding in macrophages.

## 3 Signaling pathways activating NF-κB

NF-κB is a central factor in orchestrating the responses of an organism or a cell to all different kinds of stress or threats. Hence, it can be activated by a great variety of stimuli, including pathogens such as bacteria (19), viruses (20), and even parasites (21), as well as physical stress, such as heat (22), cold (23), ionizing or UV-irradiation (24) or mechanical shear forces (25). The multiple pathways leading to activation of NF-κB can also originate from chemical or metabolic stress or include noxious crystals or deposits, for example, from cholesterol, uric acid or asbestos fibers (26). Furthermore, even the depletion of important factors for a given cell type can result in upregulation of NF-κB, as shown after androgen depletion in prostate epithelial cells (27).

**Figure 3:**
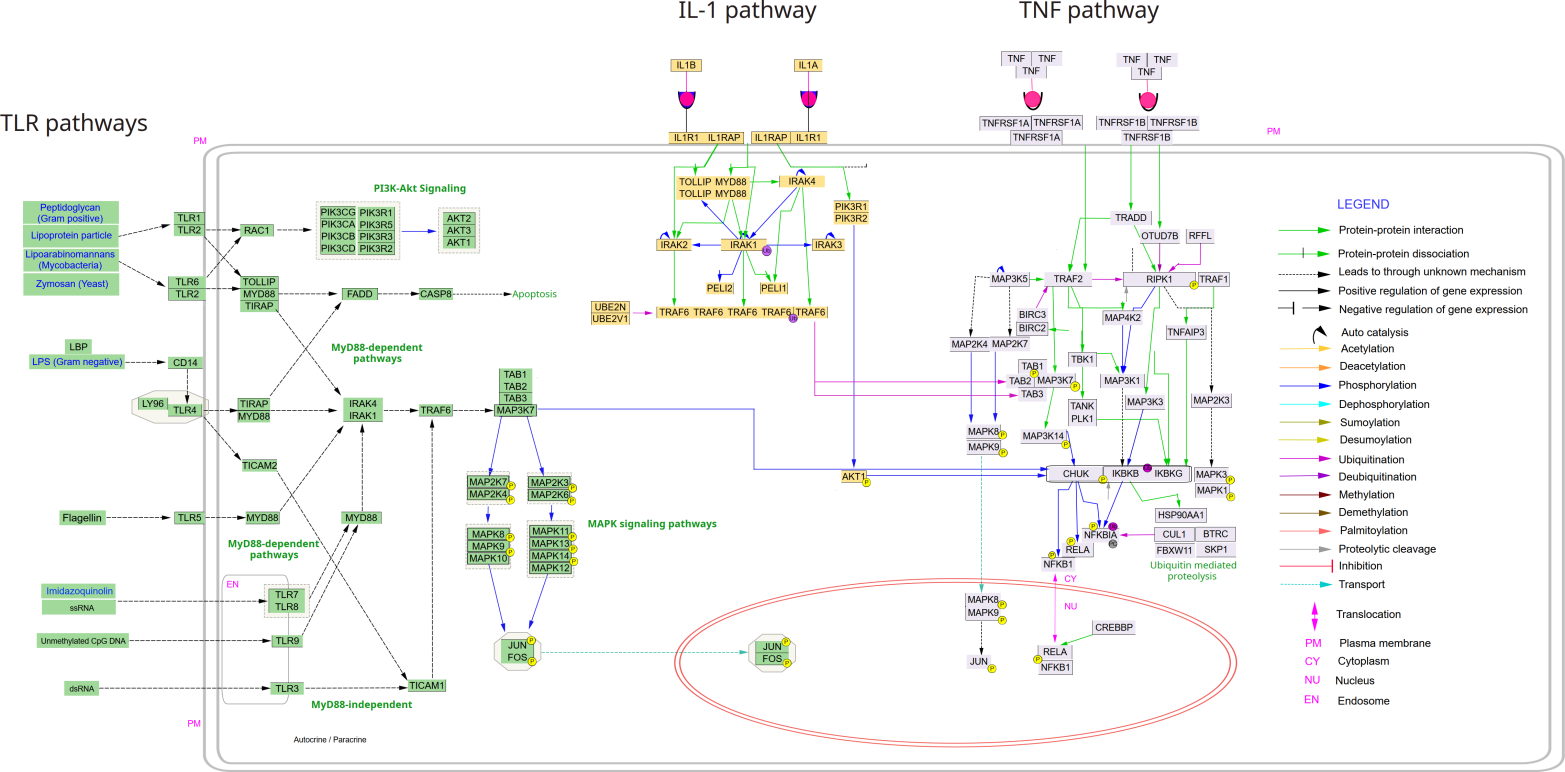
Canonical NF-κB signaling pathways from TLRs, TNFα and IL-1. The following pathways were deduced from Wikipathways (via Cytoscape) and manually curated and merged in Inkscape: *TNF alpha signaling pathway*; *IL-1. signaling pathway* and *Toll-like receptor signaling pathway*. The Cytoscape file with the three pathways is available upon request to the authors.

The signaling pathways that activate NF-κB have been grouped in several classes, although it has to be stated that some of the signaling processes are significantly overlapping and often dynamically interconnected (28), so that a clear separation of the pathways is often impossible. Nevertheless, it seems meaningful to group the activation pathways into certain categories, which share some common features. The signaling cascade that has first been elucidated with greater accuracy is the so-called canonical pathway, which is triggered by TNFα. Since it shares many similarities and signaling molecules with other inflammatory stimuli, such as IL-1β or lipopolysaccharide (LPS), the term “canonical pathway” has been extended to these as well (**Figure 3**). TNFα is a trimeric ligand, which is supposed to cause a trimerization of its receptors (TNFR1 and TNFR2), creating a signaling surface on the inner leaflet of the membrane, which results in the recruitment of adaptor proteins. Some of these proteins are ubiquitinated *via* K63- or K11-linked polyubiquitin chains, or *via* linear ubiquitination (29–32), generating binding platforms for additional signaling molecules, including kinases, which are activated – and which can propagate and probably amplify the signal to downstream kinases – a process, which includes auto- or transphosphorylation events. All these different pathways seem to converge on the level of an enzyme complex consisting of two kinases (IκB kinases: IKKα or IKK1, and IKKβ or IKK2) and an accessory protein (IKKγ or NEMO) (33–35). The IκB kinases are named after their primary function of phosphorylating inhibitory molecules (IκBs) or inhibitory protein domains of the NF-κB members p105 and p100 on two adjacent serine residues, which trigger the attachment of K48-linked polyubiquitin chains that catalyze recruitment by the proteasome activator and degradation by the 26S proteasome. A common theme of all canonical NF-κB activation pathways seems to be the crucial dependence on the IKK2 subunit of the enzyme complex.

**Figure 4:**
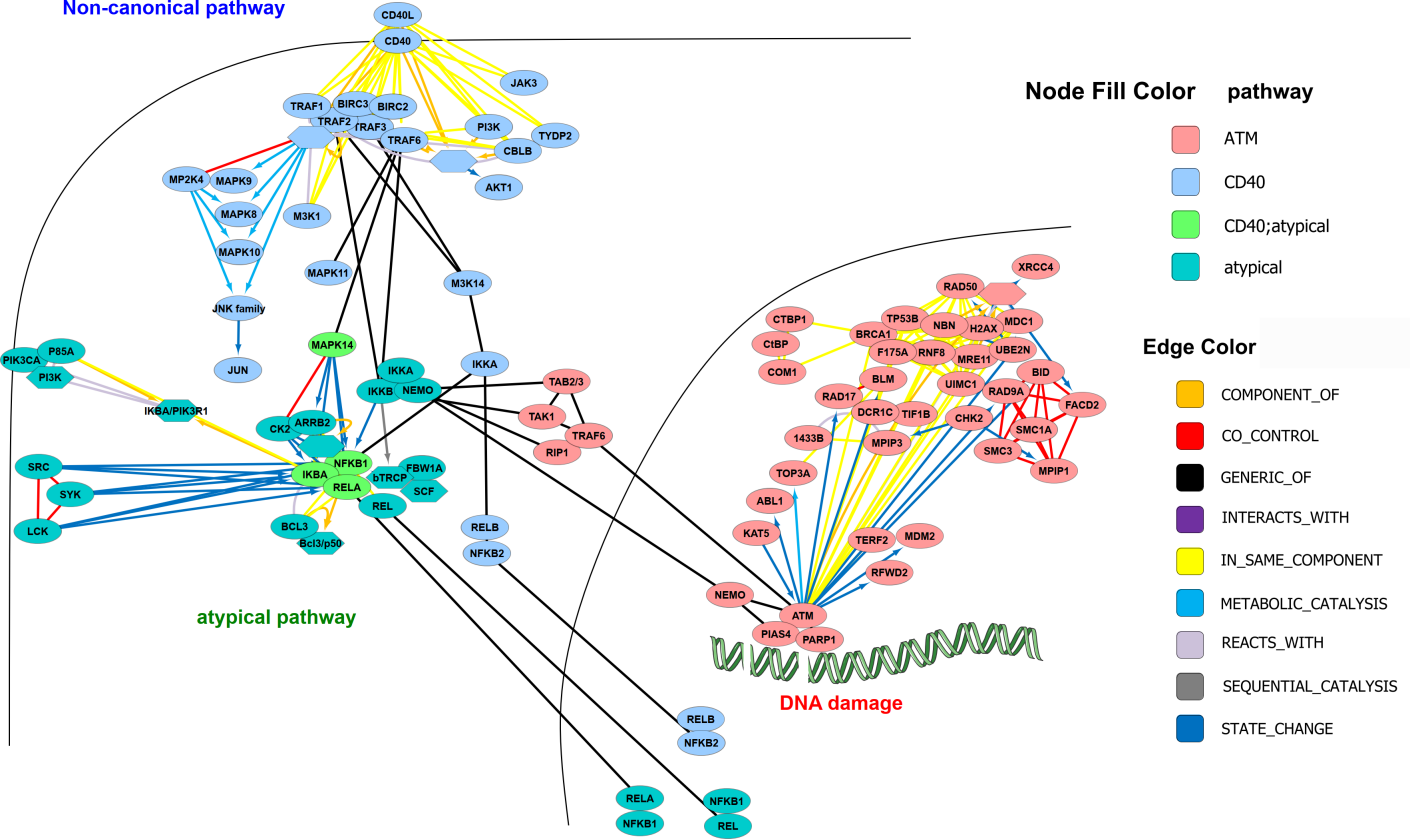
Alternative and atypical NF-κB activation pathways. The following pathways were derived from https://www.pathwaycommons.org: *CD40/CD40L signaling*, *ATM pathway* (DNA damage) and *Atypical NF-kappaB;* downloaded in BIOPAX format and imported to Cytoscape (with SIF-model mapping), where they were merged and manually curated. Nodes were arranged according to subcellular localization and additional edges as known from the literature were added. The colors of the nodes correspond to the pathways.

Alternative pathways of NF-κB activation exist, which depend on IKK1 – and which are triggered by signals such as CD40-ligand, lymphotoxin β or BAFF, requiring phosphorylation of IKK1 by NF-κB inducing kinase NIK (36, 37) (**Figure 4**). However, the various roles of IKK1 are still not clarified in detail. On one hand, it has been shown to be crucial for B-cell maturation and formation of secondary lymphoid organs (38) on the other hand, NF-κB dampening functions have been reported as well (39, 40). Moreover, it has been demonstrated that IKK1 is dispensable for IKK2 and NF-κB activation by inflammatory cytokines (41). A key question seems to be, whether IKK1 acts in complex with IKK2 and NEMO, or as homodimer (42). Furthermore, little is known about the dynamics of the IKK complex. Observations that IKK1 can shuttle to the nucleus (13), while IKK2 remains in the cytosol imply that the IKK complex can dissociate and that each IκB kinase has functions that are independent from the IKK-complex. This has been shown, for example, by reports that demonstrate that IKK1 can function as histone kinase (43) – and that it can influence the stability of c-Myc in the nucleus (44).

Apart from the so-called alternative pathway(s) of NF-κB activation, running *via* the NIK-IKK1 axis, there are other activation pathways, e.g., triggered by DNA-damage and transmitted *via* nuclear NEMO, which is then translocated to the cytosol and activating the IKK-complex (45) Additionally, pathways designated as “atypical” can originate from hypoxia or UV irradiation and act in an IKK-independent manner (33) (**Figure 4**).

An important aspect of NF-κB activation and signaling is the presence of negative regulators, which dampen or counteract the pathway. These can be present constitutively, but in many cases, they are also target genes of NF-κB, which initiate a negative feedback loop. Apart from IκB molecules themselves, which are degraded upon NF-κB activation but generally re-synthesized upon NF-κB activation, there are various signaling molecules influencing, for example, the state of activating polyubiquitin chains. These include A20 and CYLD molecules with deubiquitinase activity preferentially for the NF-κB activating K63- and K11-linked polyubiquitin chains, as well as Otulin, which degrades M1-linked ubiquitinations (46). A20 in addition harbors an E3 ligase activity, which catalyzes the attachment of degradative K48-linked chains on NF-κB activating molecules such as RIPK1, thereby functioning as ubiquitin-editing molecule shifting the balance from NF-κB activation towards degradation of NF-κB activators (46, 47).

## 4 Monocytes

Monocytes are produced in the bone marrow from myeloid precursors and circulate in the human blood at a concentration of 200-800 cells per microliter, accounting for approximately 2% - 8% of all white blood cells. Human monocytes can be subdivided into classical (~85%), intermediate (~5%), and non-classical (~10%) subsets, which are characterized by their surface expression of CD14 (LPS co-receptor) and CD16 (FcgRIII) (48). Classical monocytes (CD14^++^ CD16^-^) expressing high levels of CD14 and basically no CD16 are responsible for phagocytosis, tissue infiltration and promote inflammatory and pro-apoptotic signaling. Non-classical monocytes (CD14^+^ CD16^++^) patrol the vasculature and favor wound healing and help in resolving inflammation (49). Intermediate monocytes (CD14^++^ CD16^+^) are involved in angiogenesis and express high levels of MHC class II (50). Murine monocytes are commonly classified into Ly6C^high^ (classical) and Ly6C^low^ (non-classical) monocytes and account for less than 2% of all white blood cells (48) Knock-out of the nuclear receptor NR4A1 (Nur77) in murine monocytes and macrophages leads to a massive reduction of Ly6C^low^ patrolling monocytes which is associated with an elevated expression of TLR4 and increased phosphorylation of the NF-κB subunit p-65 (51). In LDLR- and ApoE-knockout (KO) mice, deletion of Nur77 accelerated atherosclerotic plaque development by polarizing macrophages towards a pro-inflammatory phenotype, which could be prevented by inhibition of NF-κB (51).

In an attempt to shed light on the heterogeneity of human blood monocytes, single-cell RNA sequencing of HLA-DR^+^ linage^-^ cells isolated from the blood of healthy donors was performed and led to the identification of six dendritic and four monocyte subtypes (52). Whereas the two largest clusters contained the classical and non-classical monocytes, no homogeneous population of intermediate monocytes was found. Instead, two smaller monocyte clusters could be identified, which expressed cell cycle, differentiation and trafficking genes as well as cytotoxic gene signatures, respectively (52), underlining the heterogenic nature of intermediate monocytes.

In mice, classical monocytes with a half-life below 1d convert into non-classical monocytes (half-life ~2.21d) over time, indicating that these subtypes differ in maturation and recruitment potential (53). In humans, investigating the development and kinetics of monocytes using *in vivo* deuterium-labeled glucose revealed that monocytic precursors first differentiate into classical monocytes which are retained in bone marrow for a post-mitotic maturation of approx. 38h (54). After a very short lifespan (1d) in the circulation most classical monocytes die or leave the circulation. The few remaining monocytes shift into intermediate (4.3d) and subsequently to non-classical monocytes (7.4d) (54). Consequently, non-classical monocytes show reduced telomer length and other markers of cellular senescence (17). In comparison to steady state, an acute inflammatory stimulus (such as endotoxemia) can lead to a fast release of classical monocytes in the circulation within 8h (54).

Early studies showed constitutive NF-κB activity in monocytes, that was associated with low levels of TNFα transcripts but could not be attributed to external stimuli or feedback loops (55). Current transcriptome analysis of human THP1 cells with CRISPR/Cas9-mediated NFKB1 KO revealed an increase of pro-inflammatory genes and a decrease of co-stimulatory factors in comparison to control cells (56). This data recapitulates findings from individuals with NFKB1 haploinsufficiency, which is a major immunodeficiency in Europeans (56). Alterations in basal monocytic NF-κB activation could also be involved in humans during “inflammaging”, which refers to a systemic, low-level increase of inflammation with age. In a recent study, older age was associated with decreased LPS-mediated monocytic NF-κB activation and expression of HLA-DR molecules (57). On the contrary, induction of the NF-κB pathway was observed in monocytes from patients with systemic lupus erythematosus that was attributed to monocyte activation by microparticles which serve as damage-associated molecular patterns (DAMPs) during disease development (58).

Fate-mapping studies revealed that classical monocytes are the major source for the resident monocyte-derived macrophages pool of the gut (59) and the skin (60). Upon tissue infiltration, monocytes differentiate into macrophages in the presence of macrophage colony-stimulating factor (M-CSF). M-CSF stimulates survival and differentiation of phagocytes in a protein kinase Cα- and NF-κB-dependent manner (61). Persistent nuclear translocation of NF-κB protects monocytic cells after phorbol 12-myristate 13-acetate (PMA)-induced differentiation from apoptosis (62). Inhibition of constitutive NF-κB activation by pyrolidine dithiocarbamate induces caspase 3-independent apoptosis of macrophages by transiently decreasing the mitochondrial transmembrane potential and triggering DNA fragmentation (63). This is in line with early studies that showed that embryonic macrophages of p65 knockout mice were more susceptible to TNFα-induced apoptosis (64).

Finally, monocytes have the capacity to differentiate into monocyte-derived dendritic cells (also known as TNFα-and iNOS producing (Tip-) dendritic cells (65)), which belong to a subset of dendritic cells that drastically expand during infection.

## 5 Macrophages

Macrophages constitute a heterogenous cell population with the capacity to polarize to distinct functional phenotypes. They can either derive from the differentiation of monocytes (monocyte-derived macrophages) or descend from mesodermal erythro-myeloid progenitors from the yolk sac, which spread at the onset of organogenesis (tissue-resident macrophages). The latter play pivotal functions in tissue homeostasis, have the ability for self-renewal and are independent of definitive hematopoietic stem cells, which give rise to fetal and neonatal monocytes (66). Two different pathways of erythro-myeloid differentiation have been identified: “Primitive macrophages” arise without monocytic intermediates in a c-Myb-independent manner (yolk sac macrophages and microglia). c-Myb expressing erythro-myeloid cells populate the fetal liver and generate fetal monocytes, which gradually generate the major pool of adult tissue-resident macrophages (67). Using spatiotemporal omics analysis of macrophage development, transcriptional regulators that were upregulated immediately after colonization were identified (e.g., Id3 for Kupffer cells) in mice (66). Furthermore, gene expression and imaging studies of gut-resident macrophages show distinct transcriptional profiles depending on the unique localization (e.g., proximity to blood vessels) of these self-renewing cells (68).

As macrophages are extremely plastic cells, also their epigenome, which represents a regulatory machinery of transcription factors, chromatin architecture, and histone modifications, can be altered depending on the microenvironment. In an attempt to decode stimulus-specific epigenetic reprogramming it was found that the macrophage’s capacity of epigenetic changes depends on whether NF-κB activity is oscillatory or non-oscillatory (69). For example, non-oscillatory NF-κB causes activation of latent enhancers by sustained disruption of nucleosomal histone-DNA interaction (69). By investigating the NF-κB “temporal code” in single, primary macrophages, six dynamical “signaling codons” have been identified (oscillatory vs. non-oscillatory signaling, peak amplification, duration, speed, early vs. late, total activity), which convey information about the extracellular stimulus to the nucleus (70). Oscillatory trajectories were for example found in response to cytokines, but not to pathogens. Using machine learning to apply these signaling codons on a murine model of Sjögren’s syndrome (an autoimmune disease) revealed confusion of ligand sensing and diminished stimulus specificity, potentially representing an important feature of these types of diseases (70).

Innate immune cells such as macrophages contain inflammasomes, which are multi-protein complexes comprising sensory molecules (like NLRP3, NLRC4 or AIM2) in combination with an adaptor protein (ASC) and pro-caspase-1, an inflammatory caspase, which converts pro-IL-1β and pro-IL-18 to active IL-1β and IL-18. Inflammasome activation typically requires both, a priming signal (signal 1) and an activation signal (signal 2) (71). Priming signals can be inflammatory mediators such as LPS or TNFα, which induce the expression of NLRP3 or other sensory molecules as well as pro-IL-1β *via* NF-κB binding to their promoter regions (72). This is a prerequisite for an efficient response to a second stimulus such as extracellular ATP, mitochondrial ROS, recruitment to the trans-Golgi network or K^+^ efflux (73). As an exception to the general rule, it was claimed that human monocytes only need one signal for inflammasome activation (74). It has been reported that the NF-κB activating kinase IKKβ (IKK2) is not only important for the transcriptional upregulation of inflammasome components, but also necessary for assembly of the NLRP3 inflammasome by recruiting it to the trans-Golgi network (75). This study also demonstrated a negative effect of pharmacological IKKβ inhibition on inflammasome function as assessed by caspase-1 activation, which is supported by further studies (73, 76). However, other reports rather claimed a dampening effect of the IKKβ/NF-κB axis (77, 78). Very recently, it has been demonstrated that IKKβ binds directly to NLRP3 supporting its oligomerization and assembly of the inflammasome (5, 79). The apparent discrepancies between studies suggesting an activating effect of the IKKβ/NF-κB axis on inflammasome function and those postulating a negative role, could probably be explained by differences between immediate and chronic stimulation of the related pathways, as feedback mechanisms after long-term inflammatory signaling might reverse immediate effects. Overall, NLRP3 activation is apparently an “all-or-non” event (80), where NF-κB activation might help to initiate a beneficial macrophage response that favors pathogen clearance over pyroptosis and cell death.

## 6 Macrophage polarization in health and disease

Macrophage polarization was first classified into so-called M1 or M2 phenotypes, dependent on the type of stimulation (81). M1 macrophages were considered to be generated after activation with cytokines such as TNFα or IFNγ (released by Th1 T-helper cells) or by pro-inflammatory compounds such as lipopolysaccharide and would have their main function in pathogen defense, while M2 cells would arise after stimulation with Th2-cell derived cytokines like IL-4, IL-10 and IL-13 with their primary role in wound healing and tissue repair. Soon afterwards, subtypes of M2 cells were proposed (as reviewed in (82)) and it became clear that the M1/M2-concept is an over-simplification of the various cellular states. Today, most researchers agree that these two stages are representative for a broad spectrum of macrophage phenotypes, which can be even seen as a multidimensional polarization landscape (83) and NF-κB is - to some degree - involved in all of them. M1-like macrophages (also referred to as classically activated macrophages) arise after stimulation by microbial products (e.g., LPS) and in an inflamed microenvironment (e.g., TNFα), but can be also induced by mechanical stretch *via* a mechanosensitive mechanism involving focal adhesion kinase and NF-κB (84). They are generally identified by their expression of INFγ and inducible nitric oxide synthase (iNOS). NF-κB is the major M1 macrophage transcription factor, followed by STAT1. Negative crosstalk between the crucial IκB kinase IKKβ (IKK2) and STAT1 have been reported during infection, which prevents M1 polarization and might act as negative feedback, contributing to the resolution of inflammation (85). On the other hand, M2-like polarization requires activation of the transcription factor STAT6 and leads to the production of anti-inflammatory cytokines such as IL-10 and IL-13. Thereby, M2-like macrophages have a role in helminth responses, resolution of inflammation, and wound healing. The latter, also involves NF-κB, which can be activated by macrophage IL-1β and causes autocrine, STAT6-dependent transcription of the pro-angiogenic factor VEGF-A promoting arteriogenesis (86). “LPS-tolerant” macrophages, which show decreased potential for re-stimulation, are also suspected to be M2-like macrophages and are characterized by the accumulation of (repressive) p50 NF-κB homodimers. Knock-out of the p50 NF-κB subunit prevented tolerance development and expression of M2-associated cytokines (87). In mice, knockout of NFKB1 caused chronic, progressive, low-grade inflammation that leads to premature aging due to ROS-mediated exacerbation of telomere dysfunction (88).

*In vivo*, macrophages are exposed to a great variety of different - often even conflicting - stimuli at the same time. A combination of single-cell sequencing and single-cell secretion-profiling of bone marrow-derived macrophages co-stimulated with LPS, INFγ and IL-4 shed light on the response of single macrophages to simultaneous stimulation by different cues (89). Co-stimulated macrophages showed a distinct global transcriptional program with high cell-to-cell variability, probably due to variable negative cross-regulation between the two different stimuli (89). Especially, cross-regulation between Klf4 and NFkbiz seems to cause mutually exclusive expression of IL-6 and IL-12b.

Macrophage polarization also plays an important role in tumors, with NF-κB acting as a central regulator (90, 91). Tumor-associated macrophages (TAM) are typically M2-like macrophages and exert immunosuppressive, pro-angiogenetic, and anti-inflammatory functions, thereby sustaining tumor growth (92). Thus, priming macrophages towards M1-like phenotypes is attractive for anti-tumor therapies. Knock-down of IκBα in macrophages using nanoparticle-mediated siRNA delivery was able to induce anti-tumor activity *in vitro* (93). Moreover, reduced tumor growth was observed in mice with chronically activated IKK2 in myeloid cells (94, 95). *Vice versa*, myeloid IKK2 knockout negatively affected dendritic cell maturation impairing tumor lysis by cytotoxic T cells (95). Whereas similar anti-tumor effects of NF-κB have been observed in a breast cancer metastasis model (96), myeloid IKK2 deletion demonstrated beneficial effects in colitis-induced cancer (97) and hepatocellular carcinoma (98, 99). Furthermore, it has been shown that eliminating IKK2 activity has the potential to “re-educate” macrophages towards an anti-tumor M1 phenotype (100). Thus, the IKK2/NF-κB axis can have anti- as well as pro-tumorigenic roles dependent on the type of cancer, the specific microenvironment and the duration of NF-κB activation, in particular whether it is acute or chronic.

Similarly in other settings, M1 macrophage polarization might be harmful and blocking NF-κB may represent a potential treatment strategy. This has been reported for instance for a mouse model of tumor angiogenesis, where inhibition of NF-κB with pyrrolidine-dithiocarbamate (PDTC) led to a shift in macrophage polarization from M1 towards M2, which reduced retinal neovascularization (101). Another example, where NF-κB inhibition has proven beneficial is in the case of the response of the organism to foreign bodies. Here, macrophages modulate the tissue microenvironment, thereby influencing tissue-implant integration and postoperative adhesion. Foreign bodies such as polylactide membranes prime macrophages towards the M1 phenotype, generating an inflammatory milieu, concomitant with an increase in NF-κB phosphorylation. Accordingly, coating polylactide membranes with the NF-κB (phosphorylation) inhibitor JSH-23 has been shown to result in beneficial anti-inflammatory properties of implants (102).

Macrophage polarization also plays an important role in atherosclerosis, where infiltrating monocytes differentiate predominantly into M1-like macrophages, which lose their capability of switching to M2-like cells (103). In this case, the chronic inflammatory state is usually triggered by endothelial cells, which are activated by cholesterol and lipid deposits, resulting in NF-κB-mediated expression of adhesion molecules and recruitment of monocytes. Interestingly, macrophages or macrophage-like cells located in the atherosclerotic plaque can also originate from vascular smooth muscle cells, which undergo a phenotypic transition from a contractile to a synthetic state and further on to macrophage-like cells (104, 105). It has been shown in a mouse model that inflammatory activation of endothelial cells is sufficient to drive this transition by paracrine signaling (106). The heterogeneity of cell types and states in the inflammatory microenvironment of atherosclerotic lesions has been elucidated more recently in greater detail by single-cell sequencing approaches (107).

Another specific site in the organism, where macrophage polarization has a strong impact, is the visceral fat. It is known that macrophages of an inflammatory M1-like phenotype accumulate in adipose tissue during obesity (108). These so-called adipose tissue macrophages (ATM) contribute to local/systemic inflammation and insulin resistance (109, 110). They are generated by bone marrow-derived monocytes upon infiltration, self-proliferation (111) and/or adipose tissue retention (112). ATMs of obese mice show increased nuclear translocation of p65, which increases pro-survival signaling (108). Interestingly, knock-out of the NF-κB subunit p65 in adipocytes and macrophages has been shown to decrease M1 polarization and inflammation in lean mice, but not in obese animals, where it rather increased inflammation, macrophage infiltration and M1 polarization concomitant with elevated apoptosis of adipocytes and macrophages (113). During obesity, ATM promote the release of saturated fatty acids from hypertrophied adipocytes which are able to activate NF-κB *via* binding to TLR4 thereby aggravating inflammation (114). These processes also have an impact on type 2 diabetes, as shown by a myeloid-specific knock-out of IKK2, which prevented the occurrence of insulin resistance in diet-induced obesity in a systemic manner – in muscle, fat and liver (115).

**Figure 5:**
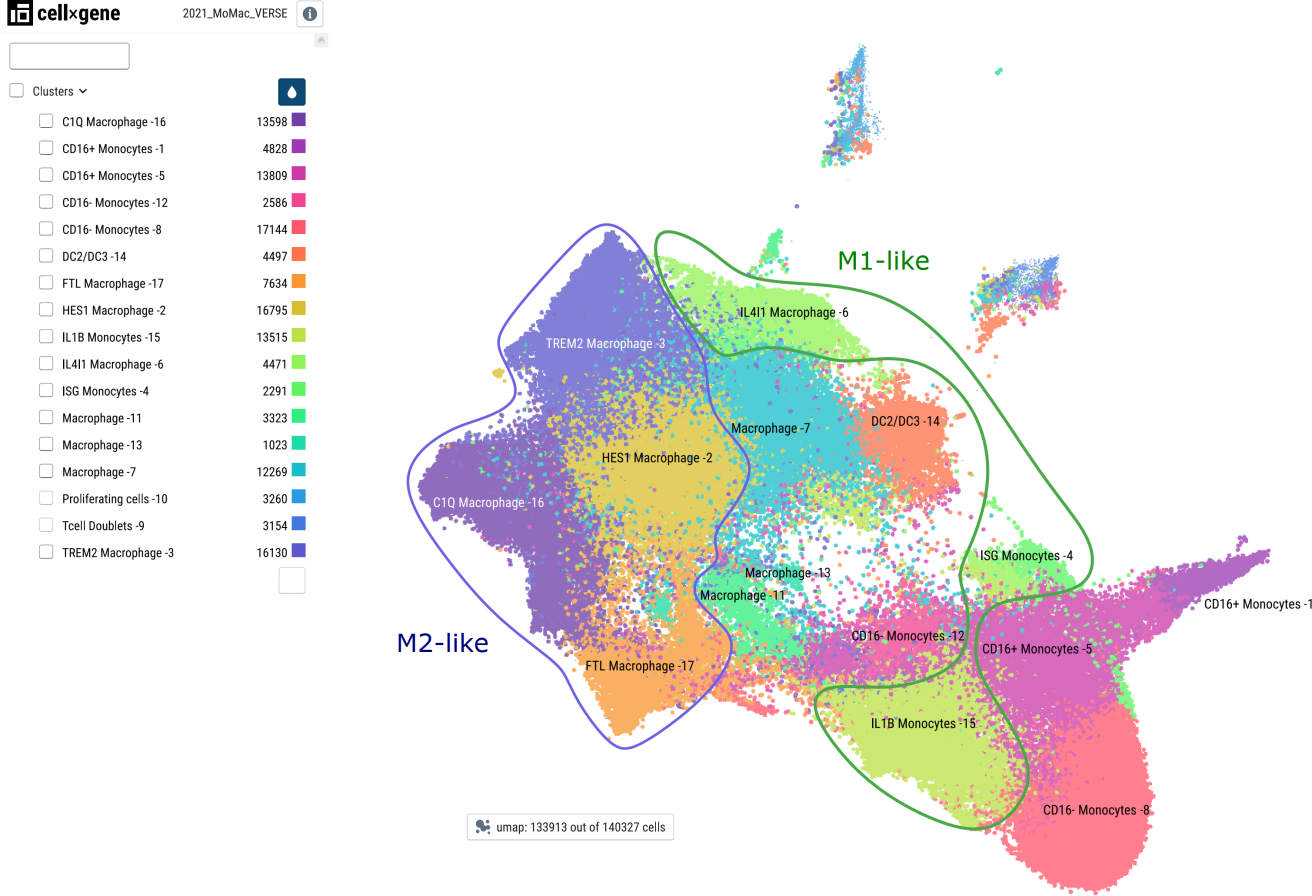
Major monocyte/macrophage subsets as defined by single-cell RNA sequencing. Major cell subsets of monocytes and macrophages were derived from the MoMac-VERSE data as described in (117) with the consent of the authors using the CZ CellxGene platform (118) with the weblink: https://macroverse.gustaveroussy.fr/2021_MoMac_VERSE/. The visualization was downloaded as pdf-file with the cell cluster names as in the publication and manually annotated for M1- and M2-like cells based on the published data (117) using Inkscape.

Lipodystrophy, basically the opposite of obesity, characterized by a deficiency of fat storage in normal (e.g., subcutaneous) adipose tissue, is interestingly similar to obesity with respect to systemic and adipose tissue inflammation including macrophage infiltration, and also leads to insulin resistance and hepatic steatosis. However, in a mouse model of this disease, myeloid deletion of IKK2 did not prevent insulin resistance, and analysis of various macrophage genes revealed clear differences to ATMs of obesity concerning gene expression patterns and response to inflammatory activation (116). Altogether, these observations indicate that macrophage states and subpopulations are very diverse – a notion, which was clearly strengthened by many different studies applying single-cell RNA-sequencing to monocytes and macrophages of different organs and physiological or pathological states, as elegantly demonstrated in a cross-tissue meta-analysis of Mulder et al. (117) (see **Figure 5**).

## 7 NF-κB target genes in monocytes and macrophages and their functions

Monocytes and macrophages are crucial for the defense of the organism against all different kinds of threats or stressors. This includes not only the response to invading pathogens, but also the reaction to damage or injury. Furthermore, they play a central role in linking unspecific innate defense mechanisms with adaptive immunity and immunological memory exerted *via* lymphocytes.

**Figure 6:**
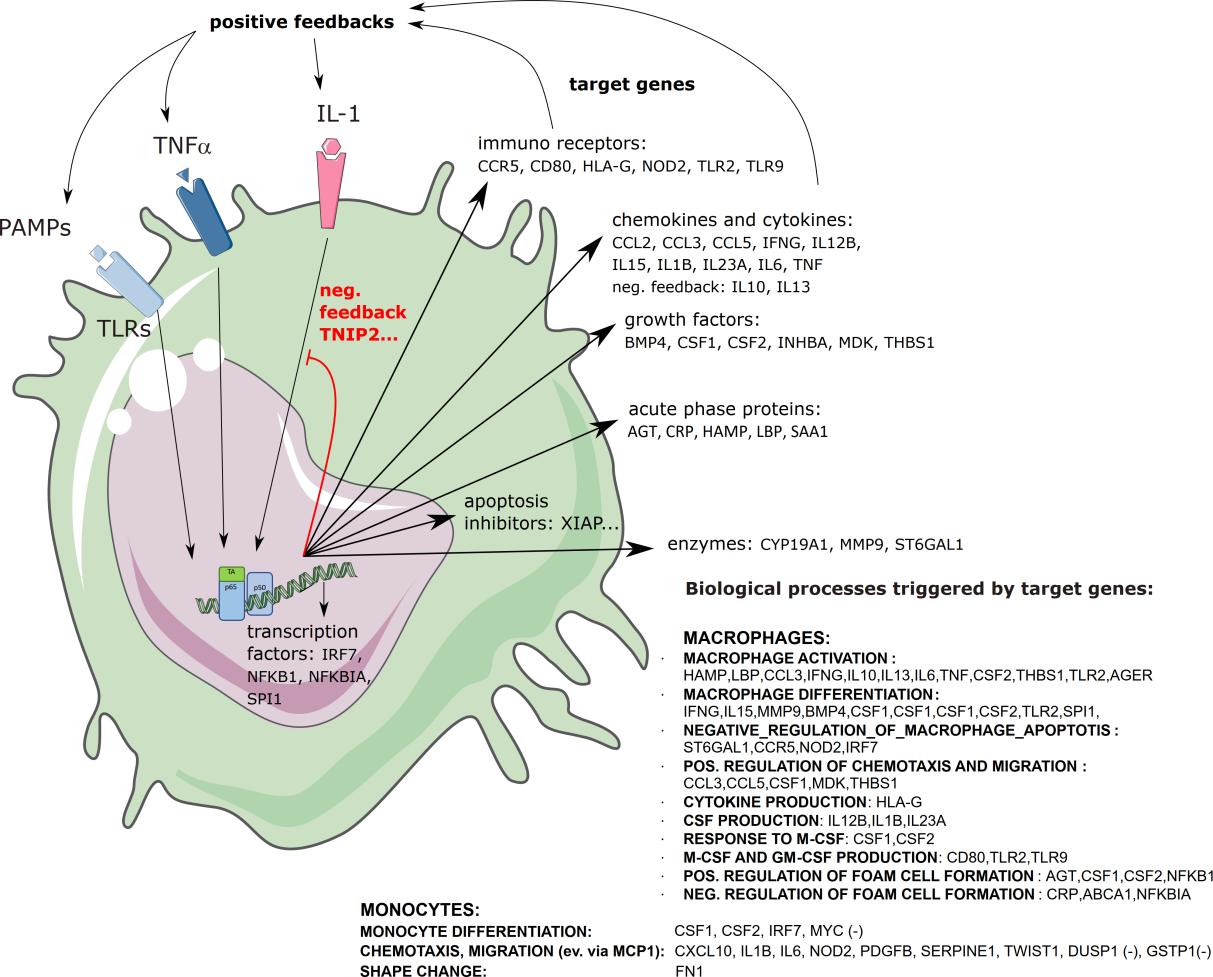
Scheme of NF-κB target genes (in monocytes and macrophages) NF-κB target genes were derived from (7) https://www.bu.edu/nf-kb/gene-resources/target-genes/). Macrophage and monocyte Gene Ontologies were retrieved using the ‘msigdbr’ package version 7.5.1, filtering for those gene sets that contained the keyword “*MACROPHAGE*” or “MONOCYTE*”. Thereafter, the intersection between the GO gene sets for monocytes and macrophages and the list of NF-κB target genes was determined.

Generally, tissue-resident macrophages of embryonic origin act as sentinels and guardians in basically all organs and tissues, where they sense the environment for the occurrence of “foreign” or dangerous structures. These resident macrophages with self-renewing, proliferative potential can be further assisted by newly recruited macrophages originating from monocytes of the blood circulation. All of these cells express a plethora of receptors recognizing pathogen-associated molecular patterns (PAMPs), as well as danger- or damage-associated molecular patterns (DAMPs). These receptors can be grouped into four classes: i) Toll-like receptors (TLRs) located on the surface of cells or in endosomal membranes; ii) NOD-like receptors (NLRs) in the cytosol as components of inflammasomes; iii) cytosolic RIG-I-like receptors recognizing mainly double-stranded RNA of viruses; and iv) cyclic GMP-AMP synthase (cGAS), which senses mislocated DNA in the cytosol (119). All of these receptors activate NF-κB and other inflammatory signaling pathways, which is commonly leading to a polarization of macrophages towards the M1-like phenotype. This initiation phase is often accompanied by positive feedback loops, such as transcriptional upregulation of inflammasome components, pro-inflammatory cytokines like IL-1, IL-6 and TNFα, induction of chemokines and their receptors as well as further expression of pattern recognition receptors (PRRs, see **Figure 6**). Moreover, colony-stimulating factors for myeloid cells (CSFs, GM-CSF) are induced, as well as anti-apoptotic genes, which increase the number and the lifespan of monocytes and macrophages. Upon inflammatory activation, the cells exert one of their main effector functions: phagocytosis of pathogens and foreign material, followed by formation of phagolysosomes and degradation. This is in many cases paralleled by formation of reactive oxygen species (ROS), which help to kill bacteria or to inactivate other pathogens. A negative side effect of this defense mechanism is the oxidative damage of own structures, which can lead to the occurrence of “altered-self” – a process that can result in a vicious cycle of chronic inflammation, and which is also a hallmark of atherosclerosis *via* formation of oxidized lipids (120). This phase of macrophage activation is furthermore characterized by transcriptional upregulation of various coagulation factors such as tissue factor (F3) on monocytes, which can be released *via* extracellular vesicles and may prime the blood for enhanced coagulation – representing a cellular link between inflammation and increased risk of thrombotic events (121). In a physiological context, this link between inflammation and coagulation factors might contribute to the process of immune-thrombosis, where invading pathogens are trapped and immobilized in micro-thrombi, preventing their spread in the organism (122, 123). After phagocytosis and inactivation of potential pathogens, the next step of the innate immune defense exerted by monocytes and macrophages is presentation of the degradation products *via* MHC class II molecules to T- and B-cells to provide a functional link to the adaptive immunity (82). Specific T-cells recognizing MHC-II bound peptides *via* their T-cell receptors are activated and secrete cytokines, which further stimulate macrophages providing a functional amplification. B-cells recognizing these antigens are re-programmed to differentiate into plasma cells producing antibodies binding specifically to these antigens, and both T- and B-cells clonally expand to support an efficient defense. Newly synthesized antibodies cover the pathogens so that their phagocytosis is facilitated *via* Fc-receptors. Besides internalization and degradation of foreign structures, macrophages also remove apoptotic cells and cell debris in a process designated as efferocytosis (124) and they degrade components of the extracellular matrix, all of which contributes to wound healing and the resolution of inflammation. This later stage of macrophage effector function is characterized by M2-like polarization. Thus, an exact timing and balance of various cellular states of monocytes and macrophages is vital for an efficient and well-controlled execution of their tasks and any imbalance might lead to pathological states.

## 8 Conclusion

In summary, hundreds of different stimuli converge at the level of NF-κB, which is a central coordinator of cellular responses of monocytes and macrophages ranging from cell polarization, activation, apoptosis, and intercellular crosstalk. Investigating cellular responses on a single cell level revealed that not every cell responds to the same stimulus in the same way. This heterogeneity is exacerbated under *in vivo* conditions when different – often conflicting – stimuli are present at the same time. We are only beginning to understand what this means for physiological and pathophysiological mechanisms. Moreover, as NF-κB is more than one component and rather represents a dynamic NF-κB signalosome it is important to unravel the unique, timing- and context-dependent roles of each component and how these different components interact. The duration and termination of NF-κB signaling is additionally important as well as the crosstalk of NF-κB with other cellular pathways and components such as the inflammasome. Beyond its action as transcriptional regulator, also epigenetic modulation by NF-κB family members will gain future attention and will add an additional layer of complexity.

## Author contributions

MM and JS structured and drafted the manuscript and wrote the final version. MD contributed to the writing. JB and JS did bioinformatics analysis and JS created the figures. All authors contributed to the article and approved the submitted version.
